# A Portable Wireless Communication Platform Based on a Multi-Material Fiber Sensor for Real-Time Breath Detection

**DOI:** 10.3390/s18040973

**Published:** 2018-03-25

**Authors:** Mourad Roudjane, Simon Bellemare-Rousseau, Mazen Khalil, Stepan Gorgutsa, Amine Miled, Younes Messaddeq

**Affiliations:** 1 Center for Optics, Photonics and Lasers (COPL), Department of Physics, Université Laval, Québec, QC G1V 0A6, Canada; mourad.roudjane.1@ulaval.ca (M.R.); mazen.khalil.1@ulaval.ca (M.K.); sgorgutsa@gmail.com (S.G.); 2 LABioTRON Bioengineering Research Laboratory, Department of Electrical and Computer Engineering, Research Centre for Advanced Materials (CERMA), Université Laval, Québec, QC G1V 0A6, Canada; simon.bellemare-rousseau.1@ulaval.ca (S.B.-R.); amine.miled@gel.ulaval.ca (A.M.)

**Keywords:** wearable system, smart textile, multi-material fibers, human breath monitoring

## Abstract

In this paper, we present a new mobile wireless communication platform for real-time monitoring of an individual’s breathing rate. The platform takes the form of a wearable stretching T-shirt featuring a sensor and a detection base station. The sensor is formed by a spiral-shaped antenna made from a multi-material fiber connected to a compact transmitter. Based on the resonance frequency of the antenna at approximately 2.4 GHz, the breathing sensor relies on its Bluetooth transmitter. The contactless and non-invasive sensor is designed without compromising the user’s comfort. The sensing mechanism of the system is based on the detection of the signal amplitude transmitted wirelessly by the sensor, which is found to be sensitive to strain. We demonstrate the capability of the platform to detect the breathing rates of four male volunteers who are not in movement. The breathing pattern is obtained through the received signal strength indicator (RSSI) which is filtered and analyzed with home-made algorithms in the portable system. Numerical simulations of human breath are performed to support the experimental detection, and both results are in a good agreement. Slow, fast, regular, irregular, and shallow breathing types are successfully recorded within a frequency interval of 0.16–1.2 Hz, leading to a breathing rate varying from 10 to 72 breaths per minute.

## 1. Introduction

Changes in breathing rate are considered to be an important indicator of major physiological and pathological instabilities, such as cardiopulmonary disease, among others [1]. The breathing rate is the number of breaths a person takes per minute. The normal breathing rate of an adult at rest is about 14 breaths/min (bpm) [2], while a newborn’s breathing rate is 37 bpm [3]. Abnormal respiration rates for adults can be categorized as hyperventilation (bpm ≥ 24), hypoventilation (bpm ≤10), or apnea [4]. The lack of tools for continuous and accurate monitoring open up huge opportunities for novel sensor development [5,6]. Existing respiration monitoring systems are usually contact-based methods. Manual methods are most commonly used for measuring breathing rate. However, these methods are unreliable and prone to error. A thermistor-based method and a technique using strain-gage pressure sensors incorporated in a strap to detect chest and abdominal movements were proposed [7,8]. Also, a capacitive sensor was utilized to determine respiratory patterns through chest expansion [9,10]. A contactless breath monitoring approach can provide an attractive alternative for monitoring breathing rate. In this line of research, infrared thermography based on wavelet decomposition [11], thermal sensors [12], thermal imaging [13], camera-based systems [14], real-time vision-based methods [15], narrow-band radars [16], ultra-wide band (UWB) radars [17], and passive radar techniques [18] were proposed. Furthermore, advances in computing technology have resulted in the development of complex algorithms for respiration monitoring. For example, a three-dimensional (3D) vision tracking algorithm has been developed to measure the breathing rate [19]. Although these methods have the advantage of being contactless, they require complex measurement equipment and signal analysis, and suffer from difficulty of usage and inaccuracy according to Al-Khalidi et al. [20]. Textile based sensors or smart textiles are other important technologies which provide more comfortable and user-friendly approaches for respiration monitoring. The sensors are integrated into the threads and fibers comprising the textiles, such as conductive yarn [21], and conductive polymers [22], or are incorporated into textiles in the form of piezoelectric sensors [23], fiber optic sensors [24], fiber Bragg grating-based sensors [25,26], multi-material fiber sensors [27], and antennas [28]. Recently, Ravichandran et al. developed a wireless system operating at a 2.4-GHz frequency to estimate the respiration rate [29]. Although the presented system was able to track the breathing of an individual with an accuracy of 1.54 bpm, the authors stressed the limitations in the detection algorithms for accurately estimating the respiration rate. Other techniques monitored breathing rate by measuring the received signal strength indicator (RSSI) in wireless networks using numerous sensors [30] or a single transmitter–receiver pair [31,32]. In both cases the signal processing requires heavy mathematical treatment and the patient is limited to lying in the bed.

To overcome all these problems, our group recently developed a novel non-invasive sensor for contactless monitoring of the breathing rate [27,33]. The sensor is a spiral antenna made from a multi-material metal–glass–polymer fiber emitting and receiving at 2.4 GHz. It is positioned on the volunteer’s chest. The sensor is integrated into a standard cotton T-shirt, and records the respiration patterns through the continuous measurement of the return loss S${}_{11}$ and the resonant frequency shift of the fiber antenna using a vector network analyzer (VNA).

In this work, we have developed a new mobile platform prototype for real-time breath detection by measuring the RSSI signal through IEEE 802.15.4 and a Bluetooth protocol at 2.4 GHz. The breath sensor consists of the spiral-shaped fiber antenna connected to a miniaturized Bluetooth transmitter. The fiber antenna is designed to transmit data over wireless communication networks at 2.4 GHz [27]. When a person wearing the T-shirt starts breathing, the antenna shape changes and so does the resonance frequency and the transmitted signal strength. The strength of the signal depends upon the chest movement. We successfully recorded breathing signals of four male volunteers (who were not in motion) with regular breathing rates at different distances from the base station. Using ANSYS HFSS software, we performed numerical breath calculations of a simulated human body (SHB) and the results support the experimental breathing rate detected by the sensor. It is important to emphasize that for medical applications, the detection of changes in a patient’s breathing pattern and respiration rate is far more important than just determining the respiration rate [34,35]. For example, respiratory distress could be diagnosed from a significant change in respiration rate or repetitive shallow breaths of an individual. Also, detection of time periods where there is no breathing signal or discontinuities in the breathing signal can help in the diagnosis of sleep apnea. Using our designed platform, we have accurately detected different classes of breathing such as: slow, fast, shallow, and irregular patterns and rates with frequencies varying from 0.16 to 1.2 Hz.

## 2. System Description

**Figure 1:**
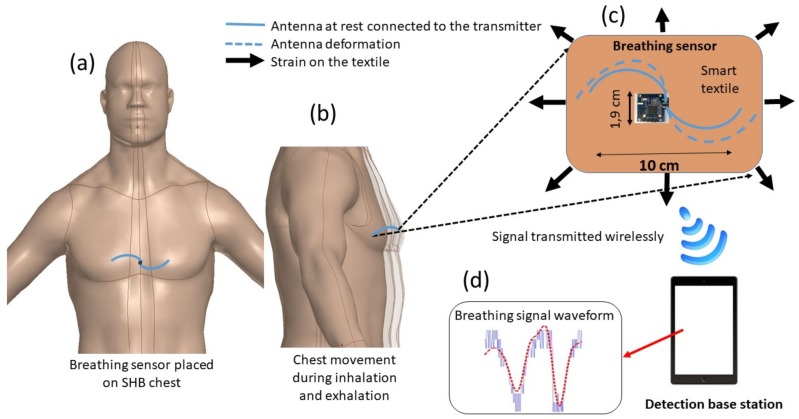
Schematic representation of the working principle: (**a**) Breathing sensor is placed on the chest of the simulated human body (SHB); (**b**) the sensor is stitched onto the elastic textile and it follows the movements of the chest wall, inducing a deformation of the spiral fiber antenna shape; (**c**) the sensor is strained by the chest movements, so the transmitted signal changes during breath; (**d**) the signal is detected wirelessly by a base station.

The proposed portable platform to monitor the respiration is composed of four parts: a spiral fiber antenna integrated into a textile, a transmission module, an energy harvesting module, and a base station. The working mechanism of the platform can be explained in three main steps as reported schematically in Figure 1: (1) the breathing sensor is stitched on an elastic T-shirt worn by a volunteer and placed horizontally in the pectoral region of the chest; (2) breathing causes significant chest movement; and (3) the transmitted signal from the sensor is sensitive to strain caused by the chest movement, so it can be used for monitoring the breathing signal. The size of the T-shirt was chosen to fit all the volunteers body shapes.

### 2.1. Fiber Antenna

**Figure 2:**
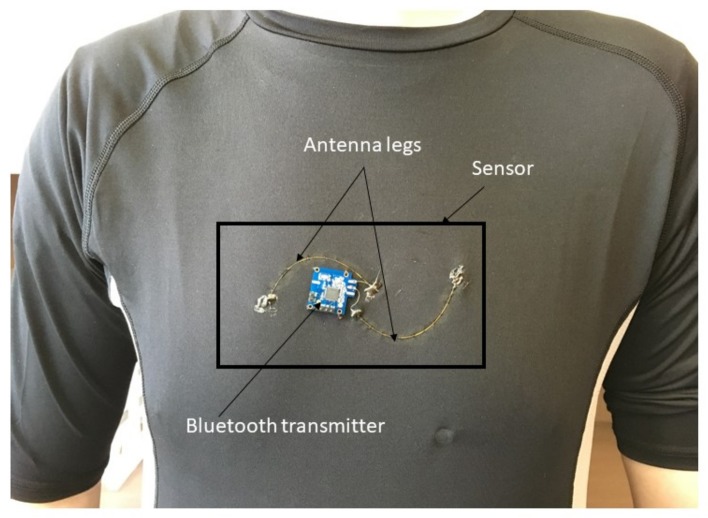
Prototype of the breath sensor made from a polyimide-coated hollow-core silica fiber antenna connected to a Bluetooth transmitter and integrated into a stretchable T-shirt.

The antenna is fabricated from multi-material fibers consisting of polyimide-coated hollow-core silica capillaries with an inner radius of 100 μm and outer radius of 181 μm, with an 18-μm-thick polyimide layer. As described in our previous work, a thin silver layer was plated on the inner surface of the hollow core [27]. An electrical DC resistance of 3.8 ± 1 Ω/cm was measured for the inner silver layer. When the length of the antenna is 10 cm, good impedance matching (50 Ω) with the standard electronic components is achieved. Using the silver-doped hollow-core fiber, we fabricated a half-turn spiral antenna as shown in Figure 2. The spiral shape of the antenna provides higher sensitivity versus the deformation of the human chest during respiration, as reported in our previous work [28].

### 2.2. Electronic System Description

The transmission module is a Bluetooth transceiver (Nordic Semiconductors SoC nRF51822) transmitting and receiving at a band rate of 250 kbps. The transmitter is a compact low energy-consuming device. It was stitched into a T-shirt and soldered to the antenna legs as shown in Figure 2.

With environmental concerns expressed in headlines worldwide, the focus is to equip the transmitter with a durable power source that would recharge itself while in use. The energy harvesting is achieved through BQ25570 chip (Texas Instruments, Dallas, TX, USA). This chip is connected simultaneously to a solar panel and a small flexible rechargeable battery. The solar energy is harvested from two small commercially available solar cells (1.2 V, 200 mW). The solar cells are connected to the rechargeable battery to recharge it when the battery voltage goes below 3 V up until it reaches 4.3 V. The output voltage is regulated by the power management chip. Efficient charging is observed when the solar cell is exposed to 3021 lux (source light radius is 3 cm and solar cell light source distance is 10 cm). The collected power is sufficient for the battery to stay charged at all times. The collected energy is 3 mW when the solar cell is closely and directly exposed to indoor light. The solar cells are used only to keep the battery charged.

The breathing pattern is extracted from the variation of the signal strength emitted at 2.4 GHz from the T-shirt’s sensor. The base station can be a smartphone, a tablet, or a computer with a Bluetooth module. Our designed base station is composed of a Raspberry Pi connected to a touch screen and a micro-controller (Quark SE C1000, Intel, Santa Clara, CA, USA). The communication between the micro-controller and the Raspberry Pi is ensured by a universal asynchronous receiver-transmitter (UART).

Once it is programmed, the transmitter acts as an advertising beacon on three channels following the Bluetooth Low Energy (BLE) protocol with a center frequency at 2.4 GHz over a narrow bandwidth of 80 MHz. When the micro-controller receives the Bluetooth signal from the transmitter sensor, it estimates the signal power through RSSI and sends all the collected values through serial communication to the Raspberry Pi. In the tested environment, this sampling method is sufficient as the breathing amplitude signal versus the noise level (SNR) is 4.9 in the best case. The sampling period is set to 20 Hz to measure the maximum breathing rate of 1.2 Hz, which satisfies the Shannon constraint. All data are displayed and plotted in real time. The breathing pattern is extracted using a home-made algorithm integrated into the Raspberry Pi. The algorithm processes data every 5 s in a window size of 40 s using a Kaiser windowing algorithm with a β coefficient of 0.6. We test the reliability of this algorithm using a Lenovo Think Pad P51 with intel core i7 and the execution time of 0.05 s. The implemented algorithm does not slow down the acquisition program when running on a separate thread with the Raspberry Pi.

Data is first filtered by a band-pass Butterworth filter with a cut-off frequency of 0.2–1.9 Hz. The center frequency of the filter is chosen with respect to the maximum and minimum breathing frequency expected from a human subject under normal conditions. The low-frequency components of the signal are filtered since the RSSI signal usually has an average value of −60 dBm. However, this value depends on the distance and the medium conditions between the emitter and the receiver. The highest breathing frequency detected by the system is 1.5 Hz. Indeed, fast breathing is not expected to be higher than 1.5 Hz even in abnormal conditions. The higher cut-off frequency (−3 dB) is then chosen at 1.9 Hz to remove all high frequencies and noises. This filter has a stable frequency response, and is used to remove the DC-component of the curve (as the usual RSSI measurements range from −45 dBm to −80 dBm), in order to smooth the curve, and attenuate all frequency components that exceed a predetermined maximum breathing frequency of 1.5 Hz. Then, a fast-Fourier transform (FFT) is applied to the treated signal in order to detect the dominant frequency, which could be associated to the breathing pattern. If none is found, the algorithm uses a continuous wavelet transform (CWT) method with Ricker wavelets to detect the number of peaks in the window. The advantage of using both the FFT and CWT methods is to accurately extract the respiration pattern, unlike in [29,31] where only FFT was used. Indeed the breathing frequency can vary inside the window, as a breathing signal can be periodic or not.

## 3. Results and Discussion

### 3.1. Design and Characteristics of the Fiber Antenna

The designed antenna was first characterized in terms of radiation performance. The key parameters that describe the antenna resonance frequency are the S-parameters, and more specifically S${}_{11}$. For best performance, a good impedance match must be observed between the antenna and the standard 50 Ω cable. The performance of the spiral antenna was experimentally and numerically analyzed using ANSYS HFSS software.

**Figure 3:**
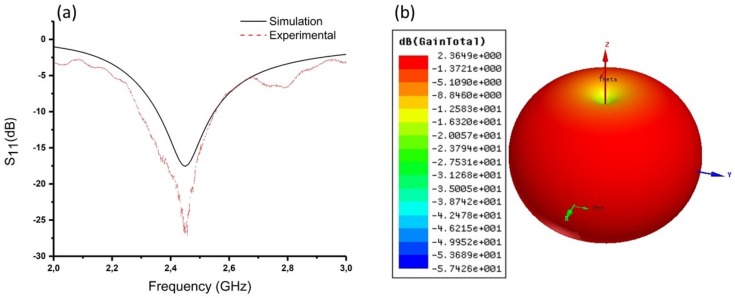
(**a**) Measured (red) and simulated (black) return loss (S${}_{11}$) for the spiral antenna. (**b**) Three-dimensional (3D) plot of the spiral antenna’s gain in the *x*, *y*, and *z* directions obtained using ANSYS software.

The antenna was first integrated into a stretchable T-shirt (available commercially). Then, it was connected electrically to either an SMA connector or to the transmitter module. Using the SMA connector, the resonant frequency shift and the return loss S${}_{11}$ were continuously measured using a VNA (HP Agilent 8722ES, HP, Palo Alto, CA, USA). The connection between the VNA and the SMA connector was performed using a 50 Ω coaxial cable. Measured and simulated S${}_{11}$ in free space are shown in Figure 3a. From this Figure, it can be seen that the antenna radiates at 2.43 GHz. Both experimental and numerical results are in a good agreement in terms of central frequency and S${}_{11}$ signal shape.

The efficiency of an antenna is related to its gain and directivity. It is defined as the power radiated relative to the power delivered to the antenna. The spiral fiber antenna gain was measured previously [27,28] at 3.45 dBi using the Friis equation approach [36]. In our case we have performed a 3D simulation for the spiral fiber antenna gain as a function of the *x*, *y*, and *z* axis using ANSYS HFSS. The result shown in Figure 3b reveals a donut-shaped radiation pattern. In this case, the transmitted power along the *z*-axis is very weak (−35.00 dB to −1.37 dB). However, in the x-y plane the radiation is maximum. The plot is very useful for visualizing in which direction the antenna radiates. Therefore, based on this result the spiral antenna gain is about 2.37 dBi, which is in good agreement with the dipole antenna gain (2.15 dBi) [37].

#### 3.1.1. Sensitivity of the Fiber Antenna in Free Space

**Figure 4:**
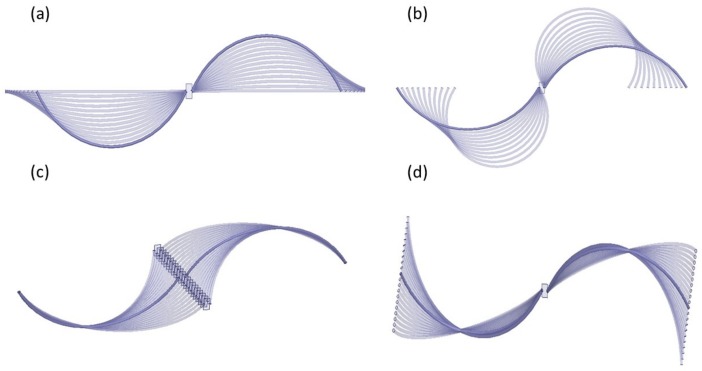
Schematic representation of the induced deformations applied to the spiral fiber antenna: (**a**) stretching, (**b**) compressing, (**c**) bending, and (**d**) folding. Simulations were performed using ANSYS.

**Figure 5:**
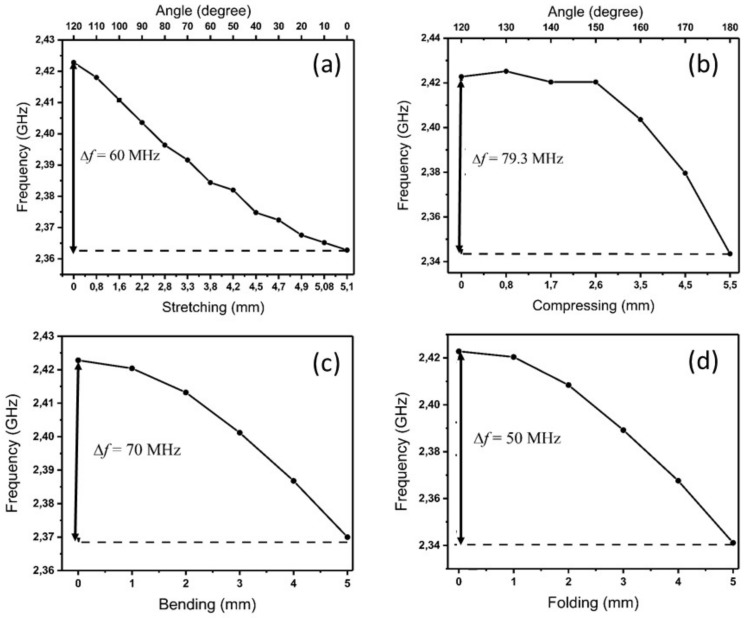
Resonant frequency shift of the spiral fiber antenna in free space as a function of the induced stretching (**a**), compressing (**b**), bending (**c**), and folding (**d**) deformations.

During respiration, inhalation is primarily due to the contraction of the human diaphragm. The contraction of the diaphragm due to the enlargement of the thoracic cavity causes the intra-thoracic pressure to fall. The latter induces lung expansion due to inspiration. However, during exhalation the diaphragm and inter-costal muscles relax. Consequently, the chest and abdomen return to the rest position. The key feature of our spiral fiber antenna design is its flexibility against stretching, shortening, twisting, or bending to detect the human chest movement. In this section, we have exposed the antenna to different induced deformation scenarios, reflecting the real environment that the antenna could face during respiration. We have studied the variation of the S${}_{11}$ signal and the resonant frequency shift as a function of the induced deformation using ANSYS FHSS numerical simulations in free space. Simulating the induced effects of the deformations, particularly the stretching and compressing, on the antenna requires an accurate control of the arc length and the angle of curvature of the spiral antenna. These are related by the equation of curvature defined as A=2π×R×(θ/360), where *A* is the arc length, θ is the angle of curvature, and *R* is the radius of the arc. It should be noted that the arc length of the spiral antenna legs is maintained constant for all the deformations. In Figure 4 we present a sketch of the fiber spiral antenna in free space subject to stretching (a), compressing (b), bending (c), and folding (d) deformations. The simulations of the S${}_{11}$ signal and central frequency shift were performed in steps of 1 mm for all the induced deformations. The behavior of the resonant frequency of the antenna as a function of the induced stretching and compressing is shown in Figure 5a,b, respectively. It can be seen that the variation of the frequency with the induced stretching deformation is linear when the antenna is elongated from (0 mm, 120${}^{\circ }$) to (5 mm, 0${}^{\circ }$), with a maximum shift of 60 MHz. For the induced compression the variation of the central frequency from (0 mm, 120${}^{\circ }$) to (2.6 mm, 150${}^{\circ }$) is relatively small (3 MHz) and drops linearly after that to reach 79.3 MHz. This frequency shift is relatively close to the one obtained for the stretching deformation. In Figure 5, we found that the central frequency shift induced by the bending (c) and the folding (d) deformations over a 5-mm length was of 50 MHz and 70 MHz, respectively. These studies allow estimation of the spiral fiber antenna sensitivities, which are summarized in Table 1 for each induced deformation.

#### 3.1.2. The Fiber Antenna’s Performance on a Simulated Human Body

The performance of the antenna is affected with the proximity of any conductive medium [38]. A simplified model of a human body with the corresponding dielectric properties was implemented in ANSYS HFSS in order to study the performances of the spiral fiber antenna. The advantage of using a SHB rather than the body phantom [39] is related to the fact that the SHB takes into account the effects of the dielectric properties and the deformation of the chest on the central frequency shift simultaneously.

During breathing, chest expansion could vary from 1 cm to approximately 3 cm. To detect this variation we have imported a complete human model inside the ANSYS environment [40]. The model is a simplified version of the exterior human body where it does not contain any organs. It is considered to react against the propagated electromagnetic field as a homogeneous model which consists of muscle only. It is well known that the human body is very complex, inhomogeneous, and built with different layers and tissues of different dielectric properties. To get realistic simulation results, we assigned the human dielectric property to this model. The dielectric properties were extracted from the federal communications commission [41], and from the Italian national research council [42]. For muscle at 2.45 GHz, the conductivity is equal to 1.73 S/m, and the relative permittivity is 52.73, with a mass density of 1040 Kg/m${}^{3}$.

**Figure 6:**
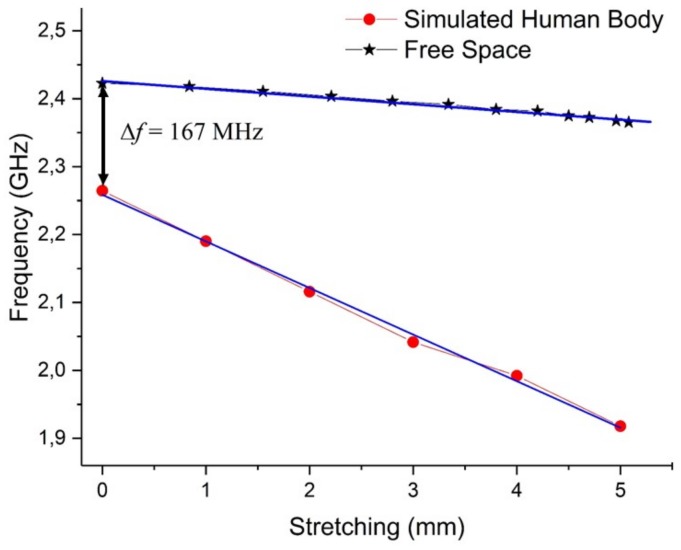
Resonant frequency shift of the textile-integrated spiral fiber antenna as a function of the induced stretch on the SHB and in free space.

We have studied the central frequency shift of the spiral fiber antenna placed on the SHB chest as a function of the induced stretching. The results are shown in Figure 6. It can be seen that when the stretch is 0 cm, the central frequency shifts by 167 MHz from the free space frequency value. This shift is induced only by the SHB’s dielectric properties. A monotonic decrease of the frequency shift is observed when the induced stretching deformation increases. This variation is similar to that observed in the free space with a clear detectable frequency difference.

### 3.2. Experimental Detection

**Figure 7:**
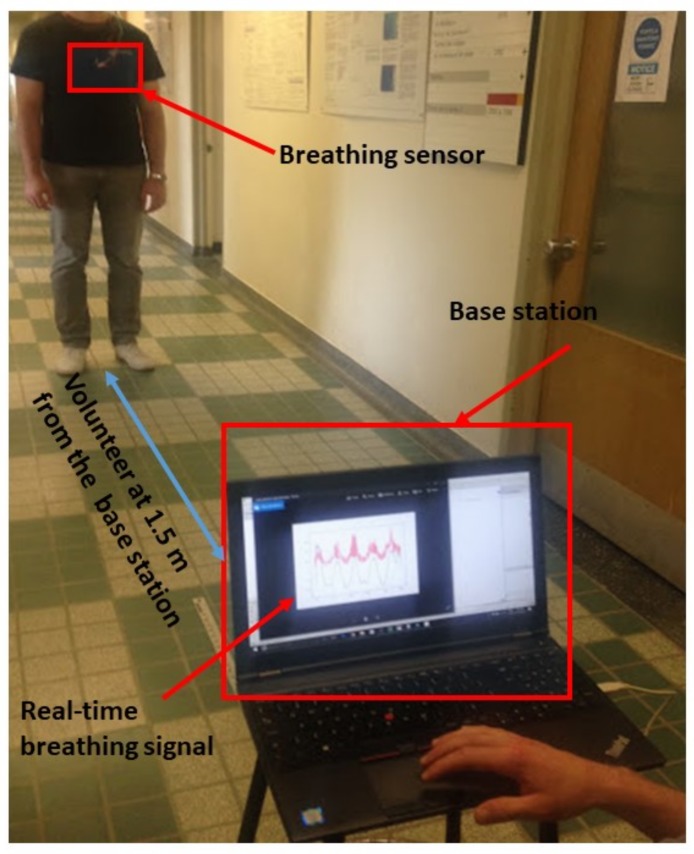
Experimental layout: volunteer wearing the smart textile and standing up in front of the base station.

We have tested the T-shirt with four healthy volunteers standing up (with no motion) in front of the base station. Breathing was performed at different rates and at different distances from the base station. The experimental layout is shown in Figure 7.

#### 3.2.1. Breathing Rate Results

**Figure 8:**
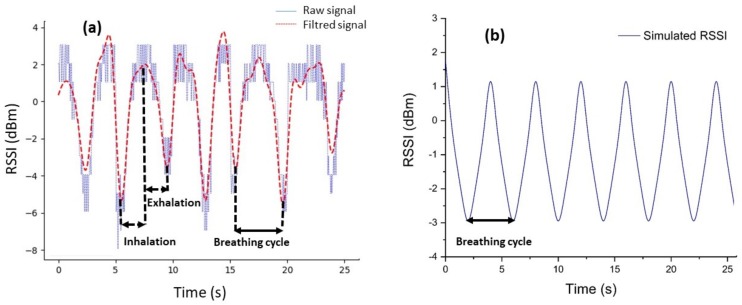
(**a**) Breathing signal from received signal strength indicator (RSSI) measurements (blue) and the filtered signal using a Butterworth filter (red). (**b**) Simulated RSSI calculated for the SHB during the breath.

A volunteer was asked to take seven regular breaths in order to detect a correlation between the respiration and the received signal at the base station. Figure 8a shows the RSSI measurements together with the filtered signal recorded for the volunteer standing at 0.5 m from the base station. The DC values from all the raw data recorded in this work were subtracted. From this Figure, although the waveform is a little bit noisy, the breathing period is clear and can be distinguished. A bandpass Butterworth filter was used to improve the signal quality. As a consequence, the inhalation and the exhalation phases in each breathing period are clearly extracted, as shown in Figure 8a. We can observe that the RSSI signal oscillates as the volunteer breathes. The breathing signal is unmistakably detected with seven breathing cycles (BCs) in 25 s. From this measurement, the inhalation and the exhalation period is estimated to be 3.57 s, which is in a good agreement with the regular BC (3 s to 5 s) reported in medical textbooks [43]. To confirm the experimental breath detection, we have simulated the transmitted signal from a spiral fiber antenna placed horizontally on the chest of the SHB standing 0.5 m from the base station. In this simulation, inhalation and exhalation times were set to 2 s for each phase. In total the breathing cycle lasts for 4 s.

In Figure 8b, we display the result of the simulation within 25 s. The RSSI signal detected during the SHB’s breath shows exactly six BCs, and each one lasts for 4 s. The signal variation follows the chest movement, hence, the breath signal of the SHB. The difference between the experimental and the simulated signal in terms of BCs is 3.57 s and 4 s, respectively. This difference could be explained by the fact that in a real environment, humans cannot maintain BCs times at precisely 4 s. Nevertheless, the simulation result and the experimental detection are in good agreement.

**Figure 9:**
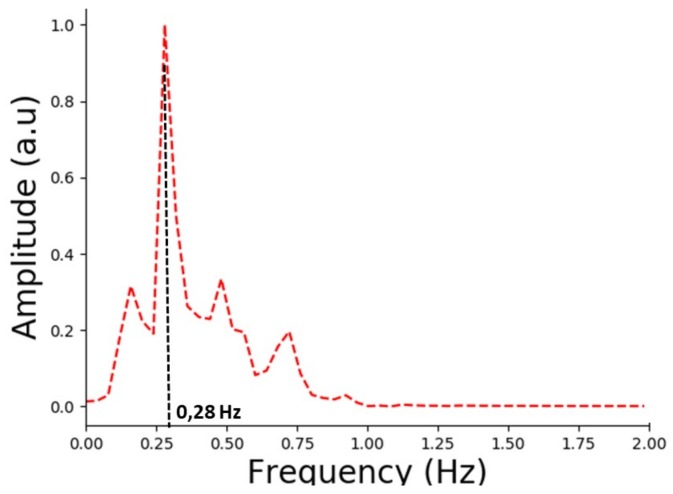
Output of fast Fourier transform for breath measurements.

To derive the dominant frequency of real breaths, we have applied an FFT. The maximum frequency at the highest amplitude corresponds to the breathing rate frequency. As can be seen from Figure 9, the measured peak is at ≈0.28 Hz, which corresponds to 16.8 bpm. This result is within the breathing rate range (12–18 bpm) for an adult as claimed in medical textbooks [43].

In Table 2, we summarize the breathing results obtained for the four volunteers who were breathing during 30 s without any restrictions on the number and rate of breaths. From this Table, we can observe that the designed sensor successfully detected different BCs for all the volunteers, corresponding to the different breathing rates chosen by each person.

#### 3.2.2. Detection of Different Breathing Patterns

**Figure 10:**
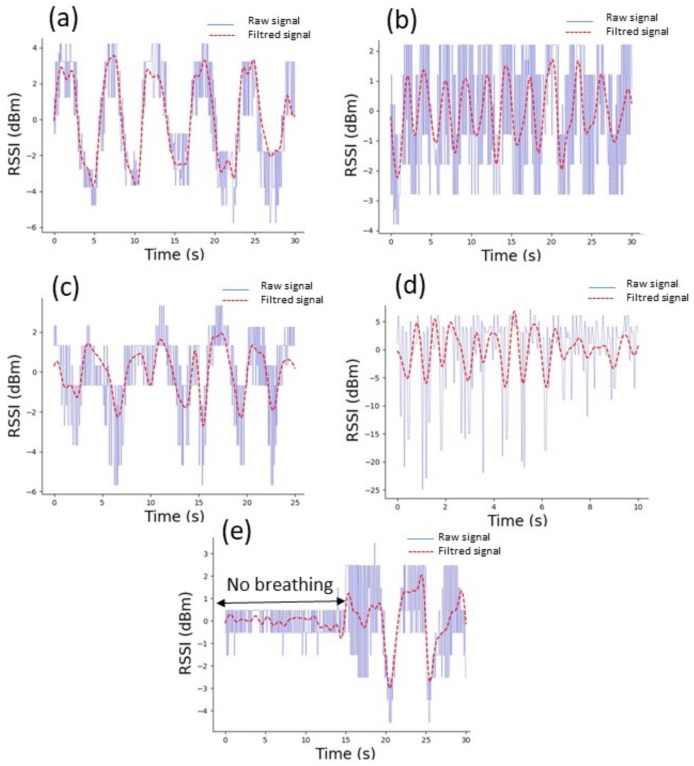
Breathing signal patterns obtained from the RSSI measurements (blue) and the corresponding filtered signals (red) for slow (**a**), shallow (**b**), irregular (**c**), fast (**d**) and a combination of no and deep long breathing (**e**).

**Figure 11:**
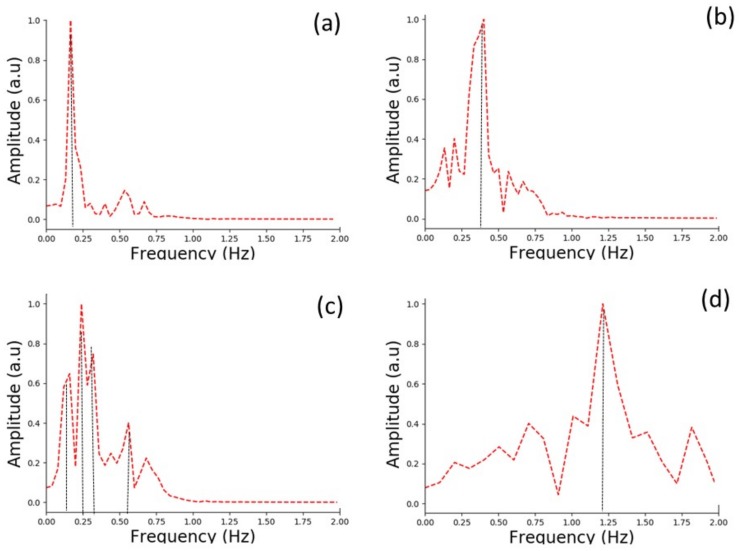
Output of Fourier transform for slow (**a**), shallow (**b**), irregular (**c**) and fast breathing (**d**).

Four different breathing cycles were performed by two volunteers standing in front of the base station. The first volunteer performed slow (a), shallow (b), and irregular (c) breathing, while the second volunteer performed fast breathing (d), and a combination of none and deep long breaths (e). For the slow breathing shown in Figure 10a, the designed sensor detected five deep BCs over 30 s. The corresponding FFT calculation provides a breathing frequency at 0.17 Hz (Figure 11a), leading to a breathing rate of 10.2 bpm.

Shallow breathing is presented in Figure 10b. The detected signal is small (two times less than the slow breathing signal) and noisy but still detectable, although it is difficult to accurately determine the inhalation and exhalation within one breath. Nonetheless, based on the filtered signal we were able to extract 11 BCs performed by the volunteer in 30 s. In this case, the dominant frequency of the breathing was measured at 0.40 Hz, as shown in Figure 11b, which corresponds to a breathing rate of 24 bpm.

It can be clearly seen that the respiration pattern in Figure 10c is irregular within 25 s. The pattern shows overlapping of different BCs. Indeed the FFT calculations reveal the co-existence of four dominating frequencies at : 0.16 Hz, 0.29 Hz, 0.32 Hz, and 0.56 Hz as shown in Figure 11c. In this case, estimating the breathing rate of the volunteer is not possible. Nonetheless, the breath sensor accurately detects the irregular breathing.

In Figure 10d we present the fast breathing measurement results. The designed system was able to detect 12 BC within 10 s. As shown in Figure 11d, the dominant frequency obtained from the FFT is 1.17 Hz, which corresponds to 70.2 bpm.

In the last case, the volunteer was asked to stop his breath for 15 s and perform a long and deep breath in the next 15 s. The result is shown in Figure 10e. During the first 15 s of the breathing waveform we can observe that the system did not detect any breathing signal. However, in the next 15 s the system recorded two clear BCs, which correspond to the two long deep breaths. The correlation between the breathing and the detected signal is clear.

## 4. Conclusions

In this paper, we have developed a new wireless communication platform to monitor in real time the breathing rate of a still individual via RSSI measurements. The system is composed of a non-invasive contactless sensor integrated into a stretchable T-shirt and a base station. The sensor is designed to operate at 2.4 GHz. It is based on a multi-material metal–glass–polymer fiber antenna in a spiral shape connected to a Bluetooth transmitter. The characteristics and the performances of the spiral fiber antenna were studied using ANSYS software. When the sensor is placed on the chest, the mechanism of breath detection is based on the central frequency shift of the fiber antenna due to the textile stretching induced by the chest movement. As a consequence, the variation of the signal amplitude during the breath is transmitted wirelessly to the base station. We have demonstrated the capability of the platform to detect the breath of four volunteers. Different breathing patterns and rates, such as slow, shallow, irregular, and fast respirations were also detected. The designed sensor is able to track a breathing with a frequency rate ranging from 0.16 to 1.2 Hz, which corresponds to a rate of 9.6–72 bpm. Numerical calculations of the simulated human breath support the experimental detection. The objective of this work is to present the concept and performance of the proposed sensor for human breathing detection. To validate our results, a comparison with gold standard equipment, such as a spirometer or a pneumotachograph, is required. This comparison should be interesting, particularly if the tests are performed on persons with respiratory problems. This should be a subject for future work.

## Figures and Tables

**Table 1:** The antenna fiber’s sensitivity for each induced deformation.

Deformation	Sensitivity (MHz/mm)
Stretching	14.2
Compressing	11.8
Bending	10.0
Folding	14.0

**Table 2:** Breathing cycle (BC), breathing frequency (BF), and breathing rate (BR) were measured for four volunteers with different weight (W) and height (H) during 30 s. The number and the rate of breath were chosen by the volunteers. BPM: breaths/min.

Volunteer	Age(years)	W (Kg)	H (cm)	BC	BF (Hz)	BR (bpm)
1	35	102	190	5.5	0.183	11
2	38	95	185	4.5	0.150	9
3	22	65	160	5.0	0.167	10
4	31	88	185	6.0	0.200	12
